# Associations between the serum triglyceride-glucose index and pericoronary adipose tissue attenuation and plaque features using dual-layer spectral detector computed tomography: a cross-sectional study

**DOI:** 10.3389/fendo.2023.1166117

**Published:** 2023-05-10

**Authors:** Yue Ma, Yanhua Zhen, Min Wang, Lingfeng Gao, Yuxue Dang, Jin Shang, Xujiao Chen, Shaowei Ma, Ke Zhou, Kai Feng, Yang Xin, Yang Hou, Chuanji Guo

**Affiliations:** ^1^ Department of Radiology, Shengjing Hospital of China Medical University, Shenyang, China; ^2^ Department of Cardiology, Shengjing Hospital of China Medical University, Shenyang, China; ^3^ Department of Cardiac Surgery, Shengjing Hospital of China Medical University, Shenyang, China

**Keywords:** pericoronary adipose tissue, dual-layer spectral detector computed tomography, coronary artery disease, fat attenuation index, triglyceride-glucose index, insulin resistance

## Abstract

**Background and aims:**

The triglyceride-glucose (TyG) index is a reliable alternative marker for insulin resistance (IR). Pericoronary adipose tissue (PCAT) can indirectly reflect coronary inflammation. IR and coronary inflammation play a key role in the development and progression of coronary atherosclerosis. Therefore, this study investigated the relationships between the TyG index, PCAT and atherosclerotic plaque characteristics to explore whether IR might lead to coronary artery atherosclerosis progression by inducing coronary inflammation.

**Methods:**

We retrospectively collected data on patients with chest pain who underwent coronary computed tomography angiography using spectral detector computed tomography at our institution from June to December 2021. The patients were grouped based on their TyG index levels: T1 (low), T2 (medium), and T3 (high). Each patient was assessed for total plaque volume, plaque load, maximum stenosis, the plaque component volume proportion, high-risk plaques(HRPs), and plaque characteristics (including low attenuation plaques, positive remodeling, a napkin ring sign, and spot calcification). PCAT quantification was performed on the proximal right coronary artery using the fat attenuation index (FAI) measured from a conventional multicolor computed tomography image (FAI_120kVp_), a spectral virtual single-energy image (FAI_40keV_), and the slope of the spectral HU curve (λ_HU_).

**Results:**

We enrolled 201 patients. The proportion of patients with maximum plaque stenosis, positive remodeling, low-density plaques, and HRPs increased as the TyG index level increased. Moreover, the FAI_40keV_ and λ_HU_ significantly differed among the three groups, and we identified good positive correlations between FAI_40keV_ and λ_HU_ and the TyG index (r = 0.319, P <0.01 and r = 0.325, P <0.01, respectively). FAI_120kVp_ did not significantly differ among the groups. FAI_40keV_ had the highest area under the curve, with an optimal cutoff value of −130.5 HU for predicting a TyG index value of ≥9.13. The multivariate linear regression analysis demonstrated that FAI_40keV_ and λ_HU_ were independently positively related to a high TyG index level (standardized regression coefficients: 0.117 [P <0.001] and 0.134 [P <0.001], respectively).

**Conclusions:**

Patients with chest pain and a higher TyG index level were more likely to have severe stenosis and HRPs. Moreover, FAI_40keV_ and λ_HU_ had good correlations with the serum TyG index, which may noninvasively reflect PCAT inflammation under insulin resistance. These results could help explain the mechanism of plaque progression and instability in patients with insulin resistance might be related to IR-induced coronary inflammation.

## Introduction

Insulin resistance (IR) is a considerable risk factor for coronary artery disease (CAD) (1), which is a major cause of death worldwide (2). IR plays a key role in the development and progression of coronary atherosclerosis. For example, IR may cause vascular inflammation (3), which is the driving force of plaque formation and a typical feature of plaque rupture, leading to acute coronary syndrome (4). The triglyceride-glucose (TyG) index is a reliable alternative marker for IR, with better predictive ability than the homeostatic model (5), and previous studies have identified a relationship between the TyG index level and adverse events in cardiovascular disease (CVD) (6–8).

Previous studies identified associations between a higher pericoronary fat attenuation index (FAI) quantified by computed tomography angiography (CTA) and coronary artery inflammation and increased CVD risk (9, 10). Additionally, coronary CTA can comprehensively assess plaque load, morphology, and composition (11). Plaque load and high-risk plaque characteristics (HRPCs), such as low attenuation plaques (LAPs), positive remodeling (PR), napkin ring signs (NRS), and spot calcification, have been identified as independent predictors of disease progression and major adverse cardiovascular events (12–14). However, the relationships between the serum TyG index and pericoronary FAI and traditional plaque characteristics have not been explored.

Double-layer spectral detector computed tomography (SDCT) is a relatively newer dual-energy CT technology that utilizes two layers of detectors to simultaneously collect low-energy and high-energy data from all patients in the same spatial and angular location, using standard CT protocols. It enables comprehensive tissue characterization by providing spectral and quantitative virtual mono-energy images (VMI) results at a wide range of energy levels, including λ_HU_ and Eff-Z (15–17). In addition to the values obtained from conventional multienergy images, the CT values from a single energy source can more accurately reflect the X-ray absorption characteristics of materials (18), which greatly improve the resolution of soft tissues and can be exploited to separate tissues with similar attenuation in conventional images (19). Recently, a study demonstrated that SDCT is suitable for coronary CTAs (20), especially using VMI reconstruction at a low-energy level (40 keV), which significantly increases the contrast of soft tissue (21–23). Moreover, other studies have shown that SDCT is more sensitive and accurate for quantifying pericoronary adipose tissue (PCAT) than conventional CT (24, 25). Therefore, we hypothesized that the PCAT index assessed by SDCT might be more helpful for distinguishing PCAT inflammation in patients with IR.

Thus, we aimed to investigate the associations between the pericoronary FAI and plaque features derived from SDCT and the serum TyG index level in patients to explore whether IR might lead to coronary artery therosclerosis progression by inducing coronary artery inflammation. The results could facilitate clinical risk stratification of patients with atypical chest pain.

## Materials and methods

### Study population

**Figure 1:**
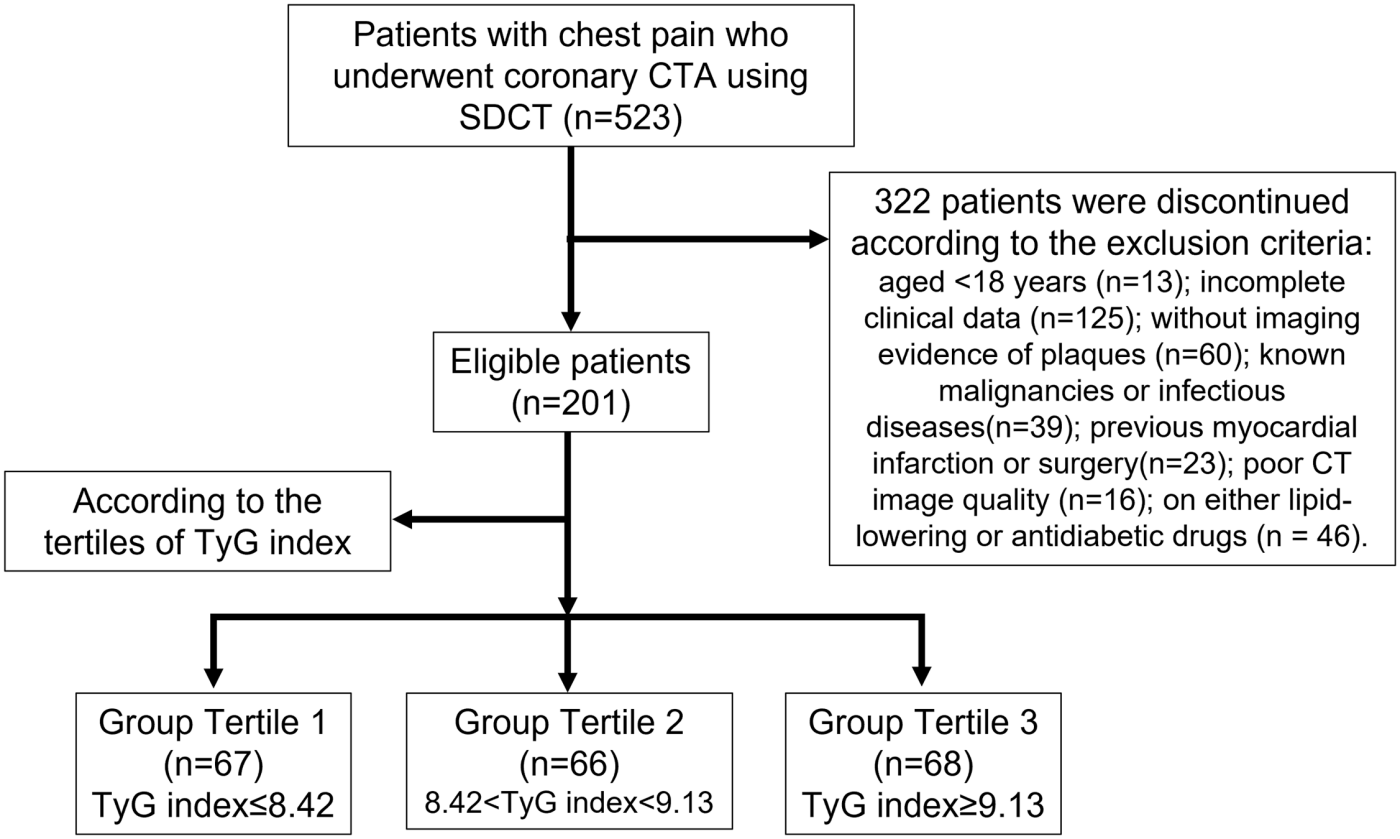
Flowchart of patient recruitment and grouping.

This retrospective study was approved by the Institutional Review Board of the Shengjing Hospital of China Medical University (No. 2021PS834K), and the requirement for informed consent was waived. From June to December 2021, we identified 523 consecutive patients with chest pain and suspected CAD who underwent coronary CTA using SDCT. The exclusion criteria were as follows: age <18 years (n = 13), incomplete clinical data (n = 125), no imaging evidence of plaques (n = 60), known malignancies or infectious diseases (n = 39), previous myocardial infarction or surgery (n = 23), poor CT image quality (n = 16), and the use of lipid-lowering or antidiabetic drugs (n = 46). Thus, we enrolled 201 patients in this study (**Figure 1**).

### Coronary CTA and reconstruction imaging protocol

All scans were performed on 64-slice SDCT (IQon Spectral CT, Philips Healthcare, Best, The Netherlands) with a prospective electrocardiogram-gated protocol (‘Step & Shoot Cardiac’). A bolus of iodinated contrast 0.8 mL/kg (iodixanol, 270 mg/mL, GE Healthcare, Ireland) was used, and flushing was performed with 20–40 mL of saline at a 4.5–5 mL/s flow rate based on the patient’s body weight. The scan parameters were as follows: 120 kVp; dose right index of 13; tube rotation time of 0.27 s; detector collimation of 64 × 0.625 mm; and slice thickness of 0.9 mm in 0.45 mm increments. The scan trigger was centered around 78% of the R-R interval, with a ±3% buffer (see the Additional Material for detailed information).

The raw data were reconstructed using a spectral iterative reconstruction algorithm. The spectral reconstruction level was set to 4 (Philips Healthcare). Next, the resulting spectral base image datasets were reconstructed into conventional polychromatic (120 kVp) images by iterative model reconstruction (Cardiac Routine Level 1, Philips Healthcare) and VMI (energy level: 40 keV and 70 keV) by spectral iterative reconstruction (Spectral Level 4, Philips Healthcare). Finally, the reconstructed images were transferred to processing workstations.

### Plaque analyses

Plaque characteristics were analyzed on polychromatic images using the IntelliSpace Portal software (version 6.5, Philips Healthcare). All coronary segments with a lumen diameter of ≥2 mm were analyzed (26). The software automatically determined the total plaque volume, total plaque burden (quantified as total plaque volume × 100%/vessel volume) (27), low-attenuation volume (≤30 HU) and percentage of plaque, intermediate-attenuation volume (31–130 HU) and percentage of plaque, high-attenuation volume (≥131 HU) and percentage of plaque (26), maximum diameter stenosis, and HRPCs. Corrections were made manually if necessary. HRPCs were assessed as follows: PR (≥1.1); low density plaque (LDP; <30 HU); spotty calcification (<3.0 mm); or an NRS (28). Plaques fulfilling at least two of these criteria were classified as high-risk plaques (HRPs).

Two cardiovascular radiologists (with 13 and 5 years of experience in cardiac imaging, respectively) were blinded to the results and independently analyzed the above parameters. The mean values of the quantitative parameters measured by the two observers were used for further analyses.

### PCAT quantification

**Figure 2:**
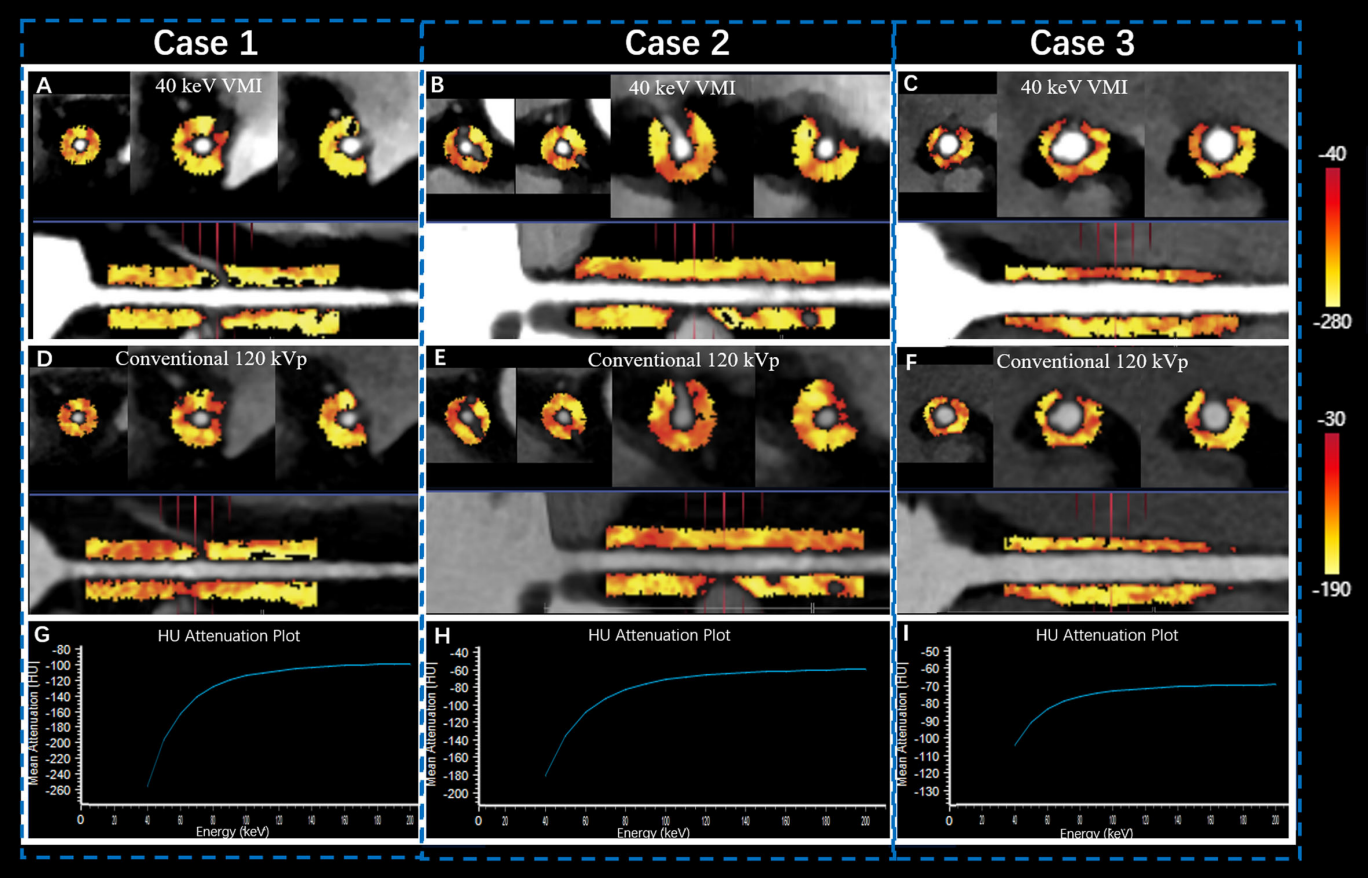
Quantitative PCAT analysis of SDCT in proximal RCA. Case 1 was a 57-year old female patient in Group T1 **(A, D, G)** with FAI_40keV_ = −159 HU, FAI_120kVp_ = −95 HU, and λ_HU_ = −2.1. Case 2 was a 67-year old male patient in Group T2 **(B, E, H)** with FAI_40 keV_ = −147 HU, FAI_120kvp_ = −94 HU, and λ_HU_ = −1.9. Case 3 was a 52-year old female patient in Group T3 **(C, F, I)** with FAI_40 keV_ = −118 HU, FAI_120kVp_ = −83 HU, and λ_HU_ = −1.37. The straightened view of PCAT of the proximal RCA in the 40 keV virtual monochromatic images **(A, B, C)** and conventional120 kVp **(D, E, F)**. G, H, I represented the corresponding spectral attenuation curve, respectively. FAI: fat attenuation index; PCAT: pericoronary adipose tissue; RCA: right coronary artery; SDCT: spectral detector computed tomography; T1-3: TyG tertile 1-3; λ_HU_: the slope of the spectral attenuation curve.

Previous studies have used the proximal right coronary artery (RCA) in PCAT analyses; thus, it represents a standardized PCAT analysis model (9, 10). Consequently, we focused on the proximal RCA (10–50 mm from the RCA opening) to standardize the PCAT analysis and perform equivalent PCAT analyses in the CTA series (**Figure 2**).

Dedicated FAI analysis software (Dr. Wise^®^ Coronary Artery CT Aided Diagnosis Software V200831, Deepwise Healthcare, Beijing, China) was used to quantify the conventional images and the 40 keV and 70 keV VMIs. Adipose tissue was identified as all voxels with attenuation thresholds of −190 to −30 HU in conventional polychromatic energy imaging, −280 to −40 HU in 40 keV VMIs, and −220 to −30 HU in 70 keV VMIs (24, 25, 29). The slope of the spectral attenuation curve (λ_HU_) was calculated as follows: λ_HU_ = (FAI_40 keV_ – FAI_70 keV_)/30. Finally, we recorded the FAIs in the proximal RCA assessed by polychromatic images (FAI_120kVp_), 40 keV VMIs (FAI_40 keV_), and 70 keV VMIs (FAI_70 keV_), as well as the λ_HU_.

### Triglyceride glucose index and other serum biomarkers

We collected data on the patients’ blood biochemical parameters, including serum total cholesterol, triglycerides (TG), high-density lipoprotein cholesterol, low-density lipoprotein cholesterol, fasting blood glucose (FBG), creatinine, and C-reactive protein (CRP) levels, within 48 h before and after the CTA examination. Per clinical practice, all blood samples were collected from the cubital vein after fasting for at least 8 h.

We calculated the TyG index as ln[TG (mg/dL) × FBG (mg/dL)/2] based on previous studies (6, 30). Then, we divided the patients into three groups based on our cohort’s TyG index tertiles (6): T1 (TyG index tertile 1: ≤8.42), T2 (TyG index tertile 2: 8.43–9.12), and T3 (TyG index tertile 3: ≥9.13).

### Statistical analyses

Datasets were analyzed using SPSS (version 23.0, IBM Corp., Armonk, NY, USA) and MedCalc version 15.2.2 (MedCalc Software Bvba, Ostend, Belgium). Continuous data were tested for a normal distribution using the Kolmogorov-Smirnov test. Normally distributed data are expressed as means ± standard deviations and were compared using analysis of variance. Nonparametric data are expressed as medians (interquartile ranges) and were compared using the Kruskal-Wallis test, and the least significant difference was used for paired comparisons. Categorical variables are expressed as percentages and were compared using the chi-squared or Fisher’s exact test, where appropriate. Correlations between the PCAT and TyG index levels were evaluated using Pearson’s correlation coefficient. The areas under the receiver operating characteristic (ROC) curve, sensitivity, and specificity were calculated to evaluate the predictive ability of FAI parameters (FAI_120kVp_, FAI40_keV_, and λ_HU_) for a high TyG index level. ROC comparisons were performed using the Delong test. Univariate and multivariate linear regressions were performed to identify independent predictors of a TyG index increase. Unstandardized regression coefficients (β, with a 95% confidence interval) and standardized regression coefficients (β) were recorded. A two-sided P-value of <0.05 was considered statistically significant.

## Results

### Clinical characteristics

A total of 201 patients were enrolled; **Table 1** summarizes the clinical characteristics of the study cohort. Among the three groups (T1, T2, and T3), body mass index, serum lipid markers, and the FBG, creatinine, and CRP levels significantly differed (P <0.05). However, age, sex, smoking history, drinking history, or the proportion of patients with hypertension did not significantly differ (**Table 2**).

**Table 1:** Demographic data.

Baseline characteristics	(n = 201)
Age (years), mean ± SD	61.3 ± 9.9(29~90)
Male, n (%)	98(48.76)
BMI, mean ± SD	24.85± 3.54
Hypertension, n (%)	119 (59.20)
Hyperlipidemia, n (%)	100 (49.75)
Diabetes, n (%)	73 (36.32)
Current smoking, n (%)	68 (33.83)
Alcohol drinker, n (%)	50 (24.88)
TC (mmol/L)	4.91± 1.10
TG (mmol/L)	1.97± 2.49
LDL (mmol/L)	2.94± 0.45
HDL (mmol/L)	1.26± 0.50
ApoA-1(g/L)	1.20± 0.27
ApoB (g/L)	0.96± 0.26
Fasting plasma glucose (mmol/L)	6.38± 4.05
CRP	3.78± 1.20
Creatinine(mg/dl)	62.27± 14.55
TyG index	8.85± 0.84

Continuous data are presented as mean ± standard deviation (SD) or median (25th, 75th percentiles). Categorical data are presented as number (%).

Apo, Apolipoprotein; BMI, body mass index; CRP, C-reactive protein; TC,Total Cholesterol; TG, Triglyceride; TyG, triglyceride glucose; HDL, High Density Lipoprotein; LDL, Low Density Lipoprotein.

**Table 2:** Clinical characteristics of patients stratified according to TyG index.

	TyG index			^+^P for overall
	Group T1 (n = 67)	Group T2 (n =66)	Group T3 (n = 68)	
Baseline characteristics				
Age (years)	62.03 ± 8.70	62.47 ± 11.86	59.31 ± 8.58	0.131
Male, n (%)	30 (44.78)	33 (50.00)	35 (51.47)	0.717
BMI	23.44 ± 3.23	24.87 ± 3.20^#^	26.22 ± 3.65^*^	<0.001
Hypertension, n (%)	41 (61.19)	41 (62.12)	37 (54.41)	0.61
Hyperlipidemia, n (%)	19 (28.36)	31 (46.97)^#^	50 (73.53)^*a^	<0.001
Diabetes, n (%)	13 (19.40)	17 (25.76)	43 (63.24)^*a^	<0.001
Current smoking, n (%)	24 (35.82)	21 (31.82)	23 (33.82)	0.888
Alcohol drinker, n (%)	18 (26.87)	16 (24.24)	16 (23.53)	0.895
Lipid markers				
TC (mmol/L)	4.65 (3.75, 5.19)	5.06 (4.26, 5.63)	5.01 (4.27, 5.74)^#^	0.035
TG (mmol/L)	0.76 (0.63, 0.96)	1.38 (1.17, 1.58)^*^	2.53 (2.04, 3.60)^*b^	<0.001
LDL (mmol/L)	2.84(2.48, 3.09)	2.92 (2.49, 3.33) ^*^	3.14 (2.78, 3.50) ^*b^	<0.001
HDL (mmol/L)	1.43(1.21, 1.83)	1.12 (0.99, 1.39)	1.03 (0.83, 1.21)^b^	<0.001
ApoA-1(g/L)	1.25 (0.99, 1.43)	1.19 (1.02, 1.32)	1.14 (0.96, 1.31)^b^	0.032
ApoB (g/L)	0.84 (0.64, 0.98)	0.96 (0.82, 1.15)^#^	1.03 (0.86, 1.17)^b^	<0.001
Risk factors				
Fasting plasma glucose (mmol/L)	5.20 (4.49, 5.64)	5.59 (5.04, 6.09)^#^	6.34 (5.53, 8.90) ^#b^	<0.001
CRP (mg/L)	2.87 (2.09, 3.99)	3.90 (3.13, 4.86)^*^	4.39 (3.38, 5.18)^b^	<0.001
Creatinine(mg/dl)	56.20(47.60, 64.90)	64.35(51.98, 76.60)^#^	62.05(50.32, 73.58)^a^	0.006
TyG index	8.04 (7.88, 8.18)	8.72 (8.60, 8.97)^*^	9.56 (9.29, 9.98) ^*b^	<0.001

Continuous data are presented as mean ± standard deviation (SD) or median (25th, 75th percentiles). Categorical data are presented as number (%).

Apo, Apolipoprotein; BMI, body mass index; CRP, C-reactive protein; HDL, High Density Lipoprotein; LDL, Low Density Lipoprotein; TC,Total Cholesterol; TG, Triglyceride; TyG, triglyceride glucose; Group T1, TyG Tertile 1; Group T2, TyG Tertile 2; Group T3, TyG Tertile 3.

^#^P< 0.05 vs. Group T1.

*P < 0.01 vs. Group T1;

a P < 0.05 vs. Group T2.

b P < 0.01 vs. Group T2.

^+^P for overall means statistical analysis among three groups.

### Plaque features and the TyG index

HRPs and high-risk features of PR and LAP were more likely to occur in group T3 than in groups T1 or T2. The maximum luminal stenosis severity was also higher in group T3 than in groups T1 or T2 (**Table 3**). Moreover, the number of HRPCs increased as the TyG index level increased (Additional **Table 1**). We also compared the number of HRPCs based on the TyG index categories, finding significant differences in the number of HRPCs among the three groups (P = 0.003; Additional **Figure 1**). In groups T1, T2, and T3, 13.43%, 16.67%, and 30.88% of lesions had ≥2 HRPCs, respectively (Additional **Table 1**, Additional **Figure 1**). However, the total plaque volume and load, calcification score, proportions of various plaque components, spot calcification, and NRS did not significantly differ between the group T3 and groups T1 and T2 (**Table 3**).

**Table 3:** Coronary CTA imaging parameters of patients stratified according to TyG index.

	TyG index			^+^P for overall
	Group T1 (n= 67)	Group T2 (n=66)	Group T3 (n=68)	
Localization of the most serious lesions, n(%)				0.078
Left anterior descending branch, n (%)	57 (85.07)	43 (65.15)	50 (73.53)	
Left circumflex branch, n (%)	1 (1.49)	7 (10.60)	6 (8.82)	
Right coronary artery, n (%)	9 (13.43)	16 (24.24)	12 (17.65)	
plaques characteristics				
maximal Diameter stenosis (%)	35.00(15.00, 55.00)	41.25 (15.00, 66.25)	57.00(30.00, 69.50)^*b^	0.001
CACS	73.31 (5.57, 371.48)	42.98 (1.76, 391.22)	105.95 (7.32, 446.65)	0.986
Total Plaque volume, mm3	70.10 (20.90, 214.70)	71.60 (14.48, 250.73)	127.10 (37.15, 354.05)	0.299
Total Plaque burden,%	1.86(0.66, 5.66)	1.74 (0.40, 6.28)	3.46 (1.02, 7.28)	0.433
High-attenuation (131HU-1300HU) plaques burden, %	38.24 (9.71, 87.82)	39.86 (8.72, 84.06)	31.93 (4.74, 69.50)	0.144
Intermediate-attenuation (31HU-130HU) plaquesburden, %	56.78 (8.45, 80.49)	54.62 (15.77, 80.80)	61.32 (31.45, 85.38)	0.358
Low-attenuation (-100HU- 30HU) plaques burden, %	1.82 (0.17, 3.67)	1.39 (0.03, 2.92)	3.59 (1.25, 8.42)	0.113
High-risk plaque characteristics				
Positive remodeling, n (%)	14 (20.90)	27 (40.91)^#^	40 (58.82)* ^b^	<0.001
Spotty calcification, n (%)	21 (31.34)	15 (22.73)	14 (20.59)	0.404
Low attenuation plaque, n (%)	2 (2.99)	5 (7.58)	10 (14.71)^#^	0.048
Napkin-ring sign, n (%)	3 (4.48)	3 (4.55)	4 (5.88)	0.914
High-risk plaque, n (%)	9 (13.43)	13 (19.70)	21 (30.88)^#^	0.043
PCAT SDCT attenuation index in RCA				
FAI_120kvp_	− 81(-87, -78)	− 82(-88, -74)	− 78(-86, -72)	0.054
FAI_40keV_	− 154(-169, -141)	−139(-157, -124)	− 123(-143, -109)^*a^	<0.001
λ_HU_	− 2.07(-2.87, -1.51)	− 1.76(-2.35, -0.93)	− 1.20(-1.57, -0.62) ^*a^	<0.001

Continuous data are presented as mean ± standard deviation (SD) or median (25th, 75th percentiles). Categorical data are presented as number (%).

CACS,coronary artery calcium score; CTA, computed tomography angiography; FAI, fat attenuation index; Group T1, TyG Tertile 1; Group T2, TyG Tertile 2; Group T3, TyG Tertile 3; RCA, right coronary artery; PCAT, pericoronary adipose tissue.

#p < 0.05 vs. Group T1; *p < 0.01 vs. Group T1.

a p < 0.05 vs. Group T2:

b p < 0.01 vs. Group T2.

^+^P for overall means statistical analysis among three groups.

### Pericoronary FAI and the TyG index

**Figure 3:**
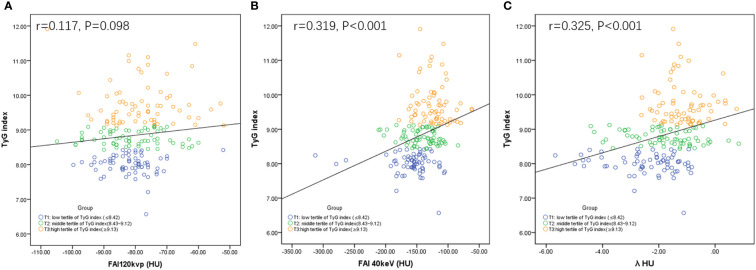
Correlations between FAI_120kVp_ **(A)**, FAI_40keV_ **(B)** or λHU **(C)** and TyG index. FAI: fat attenuation index; TyG, Triglyceride glucose; λHU: the slope of the spectral attenuation curve.

FAI_40keV_ and λ_HU_ increased as the TyG index value increased, which significantly differed among the three groups. Specifically, in group T3, FAI_40keV_ and λ_HU_ levels were higher than those in groups T1 and T2 (**Table 3**). Furthermore, the correlation analysis identified significant positive correlations between FAI_40keV_ and λ_HU_ and the TyG index (r = 0.319, P <0.01; r = 0.325, P <0.01, respectively; **Figure 3**). FAI_120kVp_ slightly increased among the three groups, but the difference was statistically insignificant, and there was no correlation with the TyG index (**Table 3**; **Figure 3**).

### FAI imaging parameters associated with a high TyG index level

**Figure 4:**
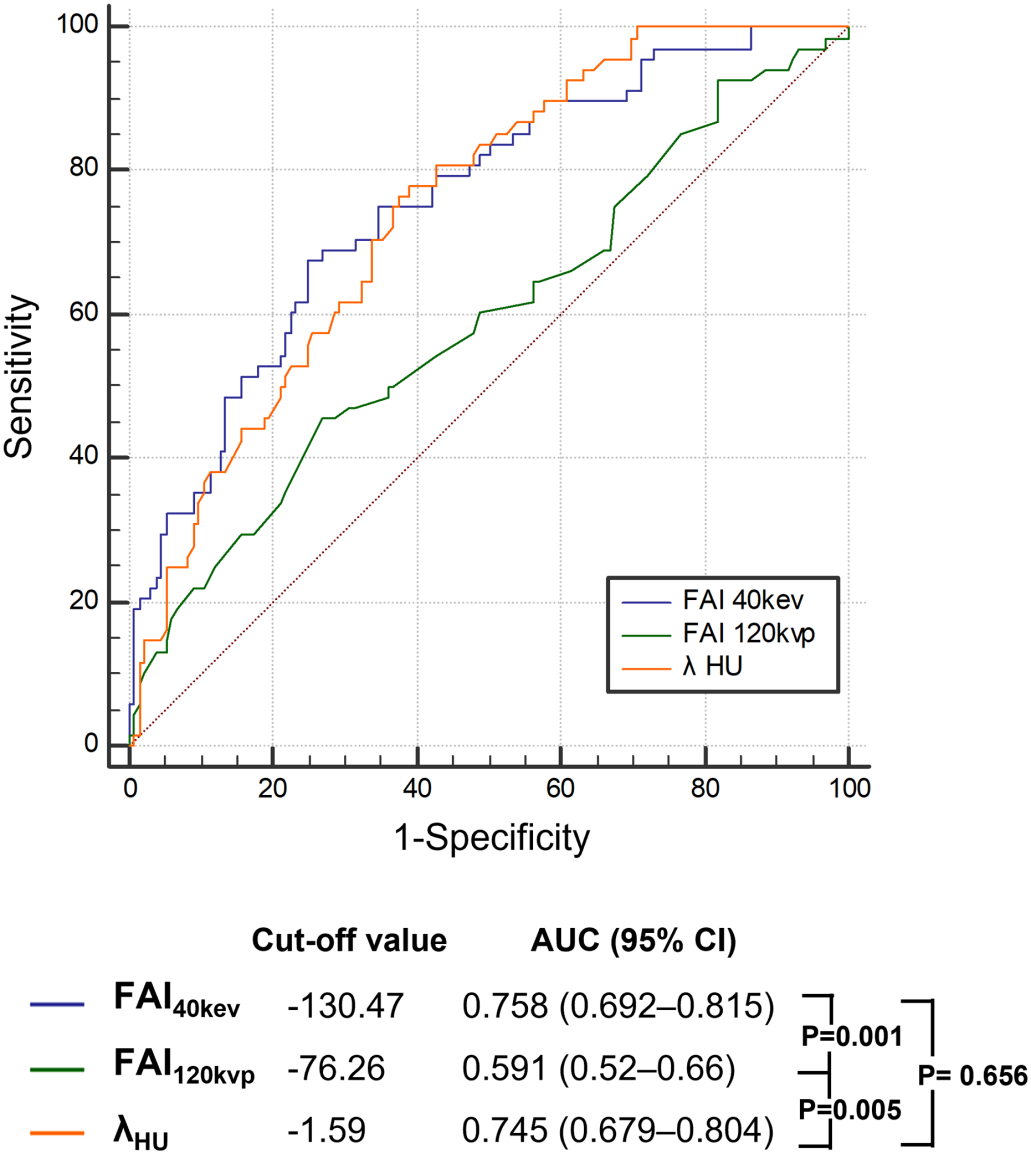
Receiver operating characteristic curve analysis showed that compared with other parameters, the FAI_40keV_ had the highest the area under curve. FAI, fat attenuation index.

The ROC curve analyses for FAI_40keV_ and λ_HU_ indicated that these parameters were superior predictors of a high TyG index level compared to FAI_120kVp_ (areas under the curve [AUCs]: FAI_40keV_: 0.758, λ_HU_: 0.745, FAI_120kVp_: 0.591; all P <0.05; **Figure 4**; **Table 4**). FAI_40keV_ had the highest AUC, with an optimal cutoff value of −130.5 HU for predicting a TyG index ≥9.13. The AUC of λ_HU_ was slightly lower than FAI_40keV_, but the two did not differ significantly.

**Table 4:** Cut-off values of FAI indices for the detection of the higher tertile of TyG index.

Parameters	Cut-off value	Sensitivity (%)	Specificity (%)	AUC (95% CI)	P
FAI_40keV_ (HU)	-130.47	67.65	75.19	0.758 (0.692–0.815)	0.036
λ_HU_	-1.59	76.47	62.41	0.745 (0.679–0.804)	0.035
FAI_120kvp_ (HU)	-76.26	45.59	72.93	0.591 (0.52–0.66)	0.044

AUC, areas under curve; FAI, fat attenuation index.

Univariate and multivariate analyses were performed to identify factors associated with a high TyG index level (**Table 5**). In the univariate analysis, maximal diameter stenosis, PR, low attenuation, HRPs, FAI_40keV_, and λ_HU_ were positively associated with an increased TyG index, whereas FAI_120kVp_ was not associated. In the multivariate analysis, FAI_40keV_ and λ_HU_ were independently and positively associated with an increased TyG index level (standardized regression coefficients: 0.117 [P <0.001] and 0.134 [P < 0.001], respectively).

**Table 5:** Uni- and Multivariate Linear Regression Analysis for the increased TyG index level.

Variables	Univariate Analysis			Multivariate Analysis			Multivariate Analysis		
TyG index	Unstandardized Coefficients*	Standardized Coefficients	P Value	Unstandardized Coefficients*	Standardized Coefficients	P Value	Unstandardized Coefficients*	Standardized Coefficients	P Value
BMI	0.075	0.318	<0.001	0.015	0.063	0.064	0.013	0.056	0.095
Hyperlipidemia, n (%)	0.69	0.413	<0.001	0.190	0.114	0.018	0.166	0.099	0.037
Diabetes, n (%)	0.722	0.416	<0.001	0.166	0.096	0.009	0.147	0.085	0.019
TC (mmol/L)	0.162	0.213	0.002	-0.047	-0.061	0.394	-0.031	-0.041	0.563
TG (mmol/L)	0.236	0,701	<0.001	0.176	0.521	<0.001	0.173	0.513	<0.001
LDL (mmol/L)	0.607	0.323	<0.001	0.062	0.033	0.318	0.077	0.041	0.209
HDL (mmol/L)	-0.645	-0.383	<0.001	-0.143	-0.085	0.031	-0.152	-0.091	0.020
ApoA-1(g/L)	-0.407	-0.133	0.061	–	–	–			
ApoB (g/L)	0.93	0.29	<0.001	0.358	0.112	0.102	0.333	0.104	0.125
Fasting plasma glucose (mmol/L)	0.1	0.482	<0.001	0.068	0.330	<0.001	0.069	0.333	<0.001
CRP(mg/L)	0.262	0.378	<0.001	0.075	0.109	0.001	0.082	0.118	0.000
Creatinine(mg/dl)	0.01	0.174	0.014	0.004	0.065	0.050	0.004	0.068	0.037
maximal Diameter stenosis (%)	0.008	0.234	0.001	0.001	0.030	0.367	0.001	0.036	0.267
Positive remodeling, n (%)	0.454	0.267	<0.001	0.139	0.082	0.035	0.131	0.077	0.046
Low attenuation plaque, n (%)	0.576	0.192	0.006	0.060	0.020	0.579	0.059	0.020	0.578
High-risk plaque, n (%)	0.526	0.258	<0.001	0.112	0.055	0.200	0.138	0.068	0.112
FAI_120kvp_	0.01	0.117	0.098	–	–	–	–	–	–
FAI_40kvp_	0.008	0.319	<0.001	0.003	0.117	<0.001	–	–	–
λ_HU_	0.237	0.325	<0.001	–	–	–	0.098	0.134	<0.001

Apo, Apolipoprotein; BMI, body mass index; CRP, C-reactive protein; FAI, fat attenuation index; HDL, High Density Lipoprotein; LDL, Low Density Lipoprotein. TC, Total Cholesterol; TG, Triglyceride; TyG, triglyceride glucose.

Note, -Unless otherwise indicated, data are β values, and data in parentheses are the 95% confidence interval.

^*^ Defined as unit increase per parameter unit change, or as specified.

## Discussion

This study’s primary finding was that an increase in the TyG index level was significantly correlated with FAI_40keV_ and λ_HU_ (represented by SDCT), and FAI_40keV_ and λ_HU_ were independently positively associated with the TyG index. Compared with the FAI obtained by conventional CT, the SDCT index may better predict a high TyG index level. Furthermore, the serum TyG index was associated with the maximum luminal stenosis severity, HRPs, PR, and LAPs.

### TyG index and PCAT FAI

IR is a potential mechanism for CAD progression (31). Therefore, measuring IR biomarkers, such as the TyG index, is valuable for stratifying risk in patients with CAD (7). Furthermore, the TyG index is a strong independent predictor of adverse clinical outcomes in patients and healthy subjects (6, 32), which may be related to vascular endothelial cell damage in an insulin-resistant state, resulting in an inflammatory response (33). Recently, FAI was proposed as a parameter reflecting vascular inflammation (9). In short, pro-inflammatory cytokines released by the inflamed artery wall can prevent lipid accumulation in perivascular adipose tissue (10). These changes and accompanying perivascular edema lead to increased perivascular adipose tissue density. Therefore, in theory, FAI would increase if blood vessel inflammation occurred. Interestingly, our results suggest a correlation between PCAT FAI and TyG index. Another possible reason for increased FAI in patients in a state of IR is related to visceral fat and small fat cells; as they become smaller, the proportion of cells increases (34), leading to higher CT attenuation and a further increase in the FAI CT value. This may explain why FAI increased more significantly at higher TyG levels.

### FAI imaging parameters by SDCT

This is the first time the relationship between TyG and PCAT indicators has been analyzed using SDCT. This study also identified significant differences in FAI_40keV_ and λ_HU_ values between groups T3 and T2 and between groups T3 and T1. More interestingly, FAI_40keV_ had a good predictive value for a high serum TyG index level. However, FAI_120kVp_ did not significantly differ among the three groups. The findings showed that discrimination between pericoronary adipose tissue was improved using SDCT at low-energy end of the spectrum (40 keV) compared to conventional polychromatic at 120 kV. It might be related to the fact that the higher attenuation differences at 40 keV gradually decreasing with increasing energy levels, and polychromatic single-energy acquisitions obtained at 120 kV (usually equivalent to monoenergy images at 70–77 keV) would yield smaller differences between tissues (35). Furthermore, conventional CT is a mixed-energy scan, meaning that the 120kVp X-ray is composed of different keV mono-energy rays. The mixed-energy attenuated images have limited ability to identify substances with high noise and beam hardening artifacts (23). Accordingly, FAI_40keV_ and λ_HU_ were significantly correlated with the TyG index. Consequently, we speculate that SDCT spectral information is superior to traditional multicolor CT in detecting and monitor subtle changes in adipose tissue composition caused by PCAT remodeling. This result is consistent with the results of Rodriguez-Granillo et al. (29) and our team (24, 25), they all found that differences of PCAT were better displayed using monochromatic imaging at low energy levels. We also observed similarities between λ_HU_ and FAI_40keV_, which may be a marker that reflects the inflammatory state of PCAT when the TyG index is elevated. These findings emphasize that SDCT-derived FAI_40keV_ and λ_HU_ may be novel sensitive imaging markers reflecting the remodeling of PCAT in the insulin-resistant state. Therefore, the relationship between vascular inflammation and IR can be preliminarily reflected by the correlation between FAI_40keV_ and the serum TyG index.

### TyG index and plaque features

Similar to pericoronary FAI, this study showed that the stenosis severity, HRP, and typical HRPCs - positive remodeling and LDP were also common in patients with an elevated serum TyG index level. Moreover, the number of HRPCs significantly increased as the TyG index level increased. This finding is consistent with previous studies (8, 36) demonstrating that the TyG index is related to the severity of coronary lesions and plaque stability. Therefore, we speculate that the degree of coronary stenosis and plaque instability may be related to the serum TyG index level.

### Clinical implications

This study verified that FAI_40keV_ and λ_HU_ by SDCT were the best CT imaging biomarkers for predicting active vascular inflammation in CAD patients with insulin-resistant state. The Cardiovascular Risk Prediction using CT (CRISP-CT) study (10) previously showed that pericoronary FAI was a prognostic index of CAD. Therefore, our results might be helpful in predicting the prognosis of CAD patients with IR. Furthermore, we found that elevated TyG index levels were more likely to have severe stenosis and HRPs, which suggested that TyG index could be used as a parameter to initiate lipid-lowering therapy in apparently healthy patients and as a selection criterion before SDCT examination to help risk stratification by coronary wall inflammation and plaque stability. In addition, these indices could help to explain the mechanism of plaque instability and atherosclerosis progression in IR patients related to IR-induced coronary wall inflammation. However, more evidence from prospective large sample studies is needed before this method can be widely used in clinical practice.

### Limitations

Despite these findings, our study had some limitations. First, this was a single-center retrospective study with a limited sample size; these results require validation in larger prospective cohorts. Second, this was a cross-sectional study, and the initial status of the participants was unknown. A longitudinal study would help reveal the relationship between the TyG index, atherosclerotic plaque characteristics, and PCAT. Finally, no other inflammatory imaging modalities (e.g., positron emission tomography) were used in this study to verify the presence of plaque inflammation. However, previous studies showed that the FAI is an effective way to detect coronary inflammation (9, 10).

## Conclusions

Chest-pain patients with elevated TyG index levels were more likely to have severe stenosis and HRPs. SDCT-based pericoronary FAI_40keV_ and λ_HU_ were independently correlated with the serum TyG index level. Therefore, FAI_40keV_ and λ_HU_ may be novel alternative imaging markers for vascular inflammation in patients with IR. These results may help explain the mechanisms related to plaque progression and instability in patients with IR, but validation in more extensive clinical trials is required.

## Data availability statement

The raw data supporting the conclusions of this article will be made available by the authors, without undue reservation.

## Ethics statement

The studies involving human participants were reviewed and approved by This study was reviewed and approved by the Shengjing Hospital of China Medical University Research Ethics Committee (No. 2021PS834K). Written informed consent for participation was not required for this study in accordance with the national legislation and the institutional requirements.

## Author contributions

YM, YH, and CG contributed to conception and design of the study. SM, KZ, MW, KF, YX, and LG conducted the data collection. YZ, YD, XC, JS, and MW conducted the image analyzing and statistical analysis. YM wrote the first draft of the manuscript. All authors contributed to manuscript revision, read, and approved the submitted version.
