# Supplementary material for: Associations between the serum triglyceride-glucose index and pericoronary adipose tissue attenuation and plaque features using dual-layer spectral detector computed tomography: a cross-sectional study
Source: Front Endocrinol (Lausanne). 2023 May 10;14:1166117. doi: 10.3389/fendo.2023.1166117 (PMC10206237; doi:10.3389/fendo.2023.1166117)
Supplement: Supplementary file 1 [file DataSheet_1.docx]

Supplementary Material

Associations between the serum triglyceride-glucose index and pericoronary adipose tissue attenuation and plaque features using dual-layer spectral detector computed tomography: a cross-sectional study

Yue Ma^1^, Yanhua Zhen^1^, Min Wang^1^, Lingfeng Gao^1^, Yuxue Dang^1^, Jin Shang^1^, Xujiao Chen^1^, Shaowei Ma^2^, Ke Zhou^3^, Kai Feng^1^, Yang Xin^1^, Yang Hou^1*^, Chuanji Guo^1*^

*** Correspondence:** Corresponding Yang Hou: houyang1973@163.com

Chuanji Guo: guocj@sj-hospital.org

# CT acquisition protocol

The coronary CTA scans were performed on a spectral detector CT scanner (IQon, Philips Healthcare, Best, The Netherlands) with prospective ECG gating technique. Using an 18-gauge catheter dual-tube high pressure syringe (Ulrich REF XD 2051), contrast media (Visipaque) (Iodixanol 270; GE Healthcare, Ireland) was injected into the antecubital vein with a flow rate of 4.5 ml/s (<80 kg body weight) or 5 ml/s (≥80 kg body weight) followed by a 20-40 ml saline flush. The total amount of contrast media was based on patient weight (patient weight × 0.8 ml / kg body weight), with the coronary CTA scan acquired 6 seconds after a threshold of 110 Hounsfield Unit (HU) had been reached in the descending aorta. The scans were performed with the following parameters: he tube voltage was 120 kVp; tube current-automatic exposure control (dose right index =13) was used to provide a balance between image quality and radiation dose; field of view =250 mm; tube rotation time =0.27 s; detector collimation =64 × 0.625 mm; matrix =512 × 512; slice thickness =0.9 mm; and increment =0.45 mm. The scan trigger was centered around 78% of the R-R interval, with a ±3% buffer used. Ahead of CT examination, patients with heart rate (HR) >70 bpm were given 25-50 mg of ß-receptor blocker (Metoprolol Succinate sustained-release tablets, AstraZeneca, Sweden) orally to reduce the HR to below 70 bpm.

# Supplementary Figures and Tables

## Supplementary Table 1. The number of HRPCs of patients stratified according to TyG index categories.

| **The number of HRPCs** | **Group** | | |
| --- | --- | --- | --- |
|  | **T1(n= 67)** | **T2(n=66)** | **T3(n=68)** |
| No HRPCs, n (%) | 36(53.73) | 31(46.97) | 15(22.06) |
| 1 HRPCs, n (%) | 22(32.84) | 22(33.33) | 32(47.06) |
| 2 HRPCs, n (%) | 9(13.43) | 11(16.67) | 19(27.94) |
| ≥3 HRPCs, n (%) | 0 | 2(3.03) | 2(2.94) |

HRPCs, High-risk plaque characteristics

## Supplementary Figure 1


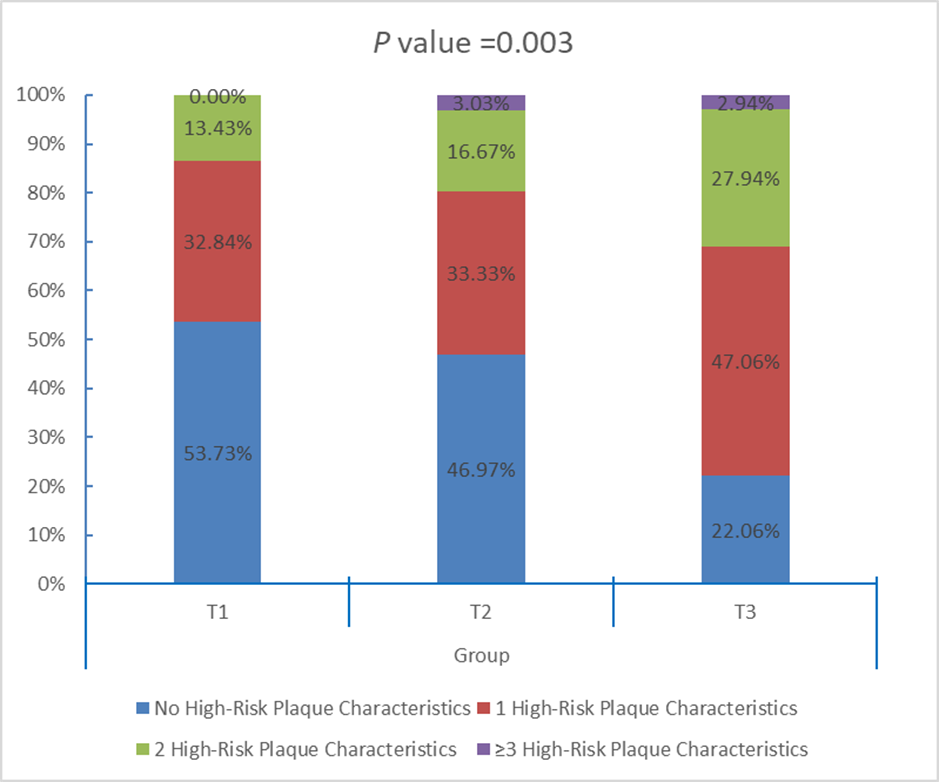


**Supplementary Figure 1.** Distribution of HRPCs According to TyG index Categories. The number of HRPCs was significantly different among different TyG index categories (Group T1 [TyG tertile 1≤8.42], Group T2 [TyG tertile 2：8.43~9.12] and Group T3 [TyG tertile 3≥9.13]). HRPCs, high-risk plaque characteristics.
